# JNK Signaling Pathway Mediates Acetaminophen-Induced Hepatotoxicity Accompanied by Changes of Glutathione S-Transferase A1 Content and Expression

**DOI:** 10.3389/fphar.2019.01092

**Published:** 2019-09-20

**Authors:** Chenxi Shi, Beili Hao, Yang Yang, Ishfaq Muhammad, Yuanyuan Zhang, Yicong Chang, Ying Li, Changwen Li, Rui Li, Fangping Liu

**Affiliations:** ^1^College of Veterinary Medicine, Northeast Agricultural University, Harbin, China; ^2^Harbin Veterinary Research Institute of Chinese Academy of Agricultural Sciences, Harbin, China; ^3^Heilongjiang Key Laboratory for Animal Disease Control and Pharmaceutical Development, Harbin, China

**Keywords:** glutathione S-transferases A1, acetaminophen, c-Jun N-terminal kinase, liver injury, hepatotoxicity

## Abstract

Acetaminophen (APAP) is an analgesic–antipyretic drug and widely used in clinics. Its overdose may cause serious liver damage. Here, we examined the mechanistic role of c-Jun N-terminal kinase (JNK) signaling pathway in liver injury induced by different doses of APAP. Male mice were treated with APAP (150 and 175 mg·kg^−1^), and meanwhile JNK inhibitor SP600125 was used to interfere APAP-induced liver damage. The results showed that JNK signaling pathway was activated by APAP in a dose-dependent manner. C-Jun N-terminal kinase inhibitor decreased JNK and c-Jun activation significantly (*P* < 0.01) at 175 mg·kg^−1^ APAP dose, and phosphorylation levels of upstream proteins of JNK were also decreased markedly (*P* < 0.05). In addition, serum aminotransferases activities and hepatic oxidative stress increased in a dose-dependent manner with APAP treatment, but the levels of aminotransferases and oxidative stress decreased in mice treated with JNK inhibitor, which implied that JNK inhibition ameliorated APAP-induced liver damage. It was observed that apoptosis was increased in APAP-induced liver injury, and SP600125 can attenuate apoptosis through the inhibition of JNK phosphorylation. Meanwhile, glutathione S-transferases A1 (GSTA1) content in serum was enhanced, while GSTA1 content and expression in liver reduced significantly with administration of APAP (150 and 175 mg·kg^−1^). After inhibiting JNK, GSTA1 content in serum decreased significantly (*P* < 0.01); meanwhile, GSTA1 content and expression in liver enhanced. These findings suggested that JNK signaling pathway mediated APAP-induced hepatic injury, which was accompanied by varying GSTA1 content and expression in liver and serum.

## Introduction

Drug-induced liver injury (DILI) is the leading cause of acute liver failure in western countries (Bernal et al., 2013). Acetaminophen (APAP), an analgesic–antipyretic, is the active component of many commonly prescribed and over-the-counter medications used to treat pain and fever worldwide (Roberts et al., 2017). Acetaminophen overdose causes acute liver failure due in part to the destruction of mitochondria as a result of increased oxidative stress followed by hepatocellular necrosis (Patterson et al., 2012). The hepatic injury model induced by APAP occupies an impregnable position in the study of liver disease. What’s more, it was reported that persistent exposure to hepatotoxic agents is clearly the primary inducer of hepatocellular carcinoma (Llovet et al., 2003). Thus, the molecular events of early hepatic injury caused by hepatotoxic agents are very important, which still need to be identified and could be a new target for precaution of hepatotoxic agents’ exposure.

C-Jun N-terminal kinase (JNK) is a member of the mitogen-activated protein kinase (MAPK) family. Numerous studies have demonstrated the importance of JNK activation in many biological processes, such as cellular differentiation, apoptosis, and stress reaction. Therefore, JNK is considered as a critical control point between the physiological and pathological status (Johnson and Lapadat, 2002; Harrison et al., 2004). C-Jun N-terminal kinase is activated by sequential protein phosphorylation through a MAPK module, namely, MAPK kinase kinase (MAP3K or MEKK) → MAPK kinase (MAP2K or MKK) → MAPK, in response to various extracellular stimuli (Karin, 1995). C-Jun N-terminal kinase is mainly activated by MKK4 (also known as MEK4/SEK1/JNKK1) and MKK7 (MEK7/SEK2/JNKK2) (Takekawa et al., 2005). Apoptosis signal-regulating kinase 1 (ASK1) was identified to activate two different subgroups of MKK, MKK4, and MKK6, and it was activated in response to oxidative stress (Ichijo et al., 1997). Oxidative stress could activate ASK1 and thereby activate ASK1-MKK4/MKK7-JNK signaling pathway. Elevated levels of p-JNK were reported to be associated with APAP-induced hepatotoxicity (Patterson et al., 2012). SP600125 is generally regarded as the specific inhibitor of JNK targeting its phosphorylation process. SP600125 was reported to suppress rat hepatocellular carcinoma by blocking tumorigenesis and tumor progression (Nagata et al., 2009). However, it remains to be elucidated that whether SP600125 has protective effect on hepatotoxicity induced by different doses of APAP.

Glutathione S-transferases A1 (GSTA1) is the main glutathione S-transferase in human liver that belongs to the family of phase II drug-metabolizing enzymes. Glutathione S-transferases A1 plays an important role in protecting the cell from acute toxic chemical assaults and canceration by relieving the toxicity of chemical substances, carcinogens, and lipid peroxide through a series of enzymatic and nonenzymatic reactions (Whalen and Boyer, 1998). It was reported that GSTA1 acted as a modulator of JNK signaling pathway by protein–protein interaction and formed GSTA1-JNK complex (Adnan et al., 2012). Once the complex dissociation occurred, JNK would be phosphorylated by MKK4, and the phosphorylation of c-Jun and c-Fos could occur following the phosphorylation of JNK. C-Jun is critical for hepatocyte proliferation and survival during liver development, regeneration, inflammation, and cancer (Hasenfuss et al., 2014). Our previous studies have demonstrated that GSTA1 is a more sensitive and more accurate indicator than alanine aminotransferase (ALT), which is a traditional biomarker of liver injury (Liu et al., 2014). Researches about GSTA1 are very important for early hepatopathy diagnosis and therapeutics. The decreased content of GSTA1 in liver while increased in serum was observed in APAP-induced hepatic injury (Shi et al., 2017). It is unclear whether JNK activation could affect liver GSTA1 during the process of DILI.

Reactive oxygen species (ROS) comprises one of the most commonly invoked cell death mechanisms during organ injury, including DILI (Jaeschke et al., 2012). The hepatotoxicity induced by APAP has been deemed to be caused by *N*-acetyl-para-benzoquinone imine (NAPQI), a cytochrome P-450–mediated intermediate metabolite, which leads to the depletion of glutathione (GSH) (Dahlin et al., 1984). Additionally, the oxidative stress caused by GSH depletion could increase the level of ROS. Hepatocyte apoptosis plays an important role in the occurrence and development of liver diseases. It was reported that JNK signaling pathway participated in the process of apoptosis (Lin, 2003). We wondered if inhibiting JNK could have effects on oxidative stress and apoptosis induced by overdose of APAP.

The primary aim of the current study is to evaluate the role of JNK signaling pathway in liver injury caused by different doses of APAP in mice. The outcome of JNK inhibition effect on APAP-induced mice hepatic injury was evaluated based on several parameters related to serum aminotransferase activities, oxidative stress, histopathological, and apoptosis examination. Additionally, the change of GSTA1 content in serum and liver was studied after inhibition of JNK to clarify whether JNK signaling pathway affects GSTA1 in liver injury.

## Materials and Methods

### Chemicals and Reagents

Acetaminophen was purchased from Aladdin Industrial Co. (Shanghai, China). SP600125 was purchased from Absin Bioscience Inc. (Shanghai, China). Alanine aminotransferase, aspartate aminotransferase (AST), malondialdehyde (MDA), superoxide dismutase (SOD), GSH, and glutathione peroxidase (GSH-Px) commercial kits were purchased from Nanjing Jiancheng Institute of Biotechnology (Nanjing, China). Mouse GSTA1 ELISA kit was purchased from Rapidbio Company (USA). Antibodies such as GSTA1 (DF6514), phospho_Ser243_-c-Jun (AF3090), phospho_Thr183+Tyr185_-JNK1/2/3 (AF3318), β-actin (BF0198), c-Fos (AF0132), phospho_Ser362_-c-Fos (AF3053), ASK1 (AF6477), phospho_Ser966_-ASK1 (AF3477), MKK4 (AF6321), and phospho_Ser80_-MKK4 (AF3321) were obtained from Affinity Biosciences (Ohio, USA). C-Jun (WL02863), Bax (WL01637), caspase-3 (WL01992a), and JNK1/2/3 (WL01295) antibodies were obtained from Wanlei Bioscience Co., Ltd. (Shenyang, China).

### Methods

#### Animals and Experimental Design

Male Kunming mice (30 days old, weighing 18–22 g) were purchased from the Central laboratory of Harbin Pharmaceutical Group Co. (Harbin, China) and housed in laboratory animal facilities under a standard 12-h light/dark cycle maintained at a temperature of 20 ± 2°C, a relative humidity of 40% to 60% and had free access to distill water and a standard rodent diet. The mice were acclimatized to laboratory condition for a week prior to experiments. All procedures involving animals complied with the National Institutes of Health Guide for the Care and Use of Laboratory Animals and the Guiding Principles in the Use of Animals in Toxicology (Pj, 2002).

First, to select the proper dosages of APAP that could cause different degrees of hepatotoxicity, the mice were randomly divided into six groups with six mice per group. After fasting for 12 h, the mice were administered with different dosages of APAP (100, 125, 150, 175, and 200 mg·kg^−1^) or saline. Twelve hours later, the mice were anesthetized (1.5% isoflurane), and blood was gained from the retro-orbicular plexus, and serum was separated for later measurement.

In order to select the proper dosage of SP600125, mice were randomly divided into seven groups after fasting for 12 h and administered with APAP (175 mg·kg^−1^) or vehicle (saline) by gavage. The mice received SP600125 (1, 2, 3, 5, and 10 mg·kg^−1^) or vehicle (DMSO–saline) by intragastric administration. After 12 h, serum was obtained for the measurements of aminotransferases.

Finally, 30 mice were fasted for 12 h and then divided into five groups randomly including control group (DMSO–saline + saline), APAP 150 group (DMSO–saline + 150 mg·kg^−1^ APAP), APAP 175 group (DMSO–saline + 175 mg·kg^−1^ APAP), SP+APAP 150 group (SP600125 + 150 mg·kg^−1^ APAP), and SP+APAP 175 group (SP600125 + 175 mg·kg^−1^ APAP). Mice were administered with APAP (150 mg·kg^−1^ or 175 mg·kg^−1^) or vehicle (saline) and then with SP600125 (2 mg·kg^−1^) or vehicle (DMSO–saline) by intragastric administration. After 12 h, serum was obtained and liver samples were also collected and stored at −80°C for further studies, and a portion of each liver sample was fixed in 10% formalin for histopathological and apoptosis analysis. Acetaminophen was dissolved in 40°C saline, and mice were given 10 mL·kg^−1^ body weight. SP600125 was dissolved in DMSO–saline solution, in which the concentration of DMSO was 2%. Mice were given 5 mL·kg^−1^ body weight, corresponding to 0.11 g·kg^−1^ DMSO.

#### Serum Biochemical and Hepatic Peroxide Assay

Alanine aminotransferase and AST activities in serum were analyzed using commercial kits according to the manufacturer’s protocols. Liver tissue samples were grinded with saline and then centrifuged. The activities of SOD and GSH-Px and the contents of MDA and GSH in the supernatant were analyzed by using commercial kits according to the manufacturer’s protocols.

#### Serum and Liver GSTA1 Analyses

Glutathione S-transferases A1 content in serum and supernatant of liver homogenates was analyzed by mouse glutathione S transferase alpha1 ELISA kit. The procedure was performed according to the manufacturer’s protocol. The assay was repeated three times.

#### Western Blotting Analyses

Liver proteins were extracted for the analysis of the β-actin, cysteinyl aspartate specific protease 3 (caspase-3), B cell lymphoma 2 associated x (Bax), GSTA1, JNK1/2/3, phospho_Thr183+Tyr185_-JNK1/2/3 (p-JNK1/2/3), c-Jun, phospho_Ser243_-c-Jun (p-c-Jun), c-Fos, phospho_Ser362_-c-Fos (p-c-Fos), ASK1, phospho_Ser966_-ASK1 (p-ASK1), MKK4, and phospho_Ser80_-MKK4 (p-MKK4) expression levels using a total protein extraction kit (Beyotime Biotech, Nanjing, China). The protein concentrations were measured using bicinchoninic acid protein assay kit (Nanjing Jiancheng Institute of Biotechnology, Nanjing, China). Liver homogenates (40 µg proteins/lane) were separated by electrophoresis using 10% to 15% sodium dodecyl sulfate polyacrylamide gel electrophoresis followed by transfer to nitrocellulose membranes. Membranes were then probed overnight at 4°C with primary antibodies. After immunoblotting with the horseradish peroxidase–conjugated secondary antibody, the immunopositive bands were detected by using enhanced chemiluminescence method using a chemiluminescence system (Bio-Rad, CA, USA). Densitometric analysis of the target bands was measured by ImageJ (National Center for Biotechnology Information).

#### Histopathological Analyses

The liver tissues from different groups were collected and fixed in 10% neutral phosphate-buffered formalin, dehydrated in graded ethanol series ranging from 50% to 100%, cleared in xylene, and embedded in paraffin. Five-micrometer-thick sections were prepared, stained with hematoxylin and eosin dye (H&E), and scanned by Leica Aperio CS2 slide scanner (Wetzlar, Germany).

#### Apoptosis Test by Hoechst 33258

Five-micrometer sections prepared according to Section 2.2.5 were stained with Hoechst 33528 dye for 5 min in a darkroom and then observed with a fluorescence microscope (OLYMPUS, Tokyo, Japan).

#### Statistical Analysis

The data were expressed as mean ± standard deviation (SD). The differences between different groups were analyzed using one-way analysis of variance. *P* < 0.01 was considered as statistically significant, and *P* < 0.05 was considered as markedly significant with the Tukey’s multiple comparison test. Statistical analyses were carried out using SPSS version 19.0 software (SPSS, Inc., Chicago, IL, USA).

## Results

### Dosage Selection of APAP and SP600125

**Figure 1:**
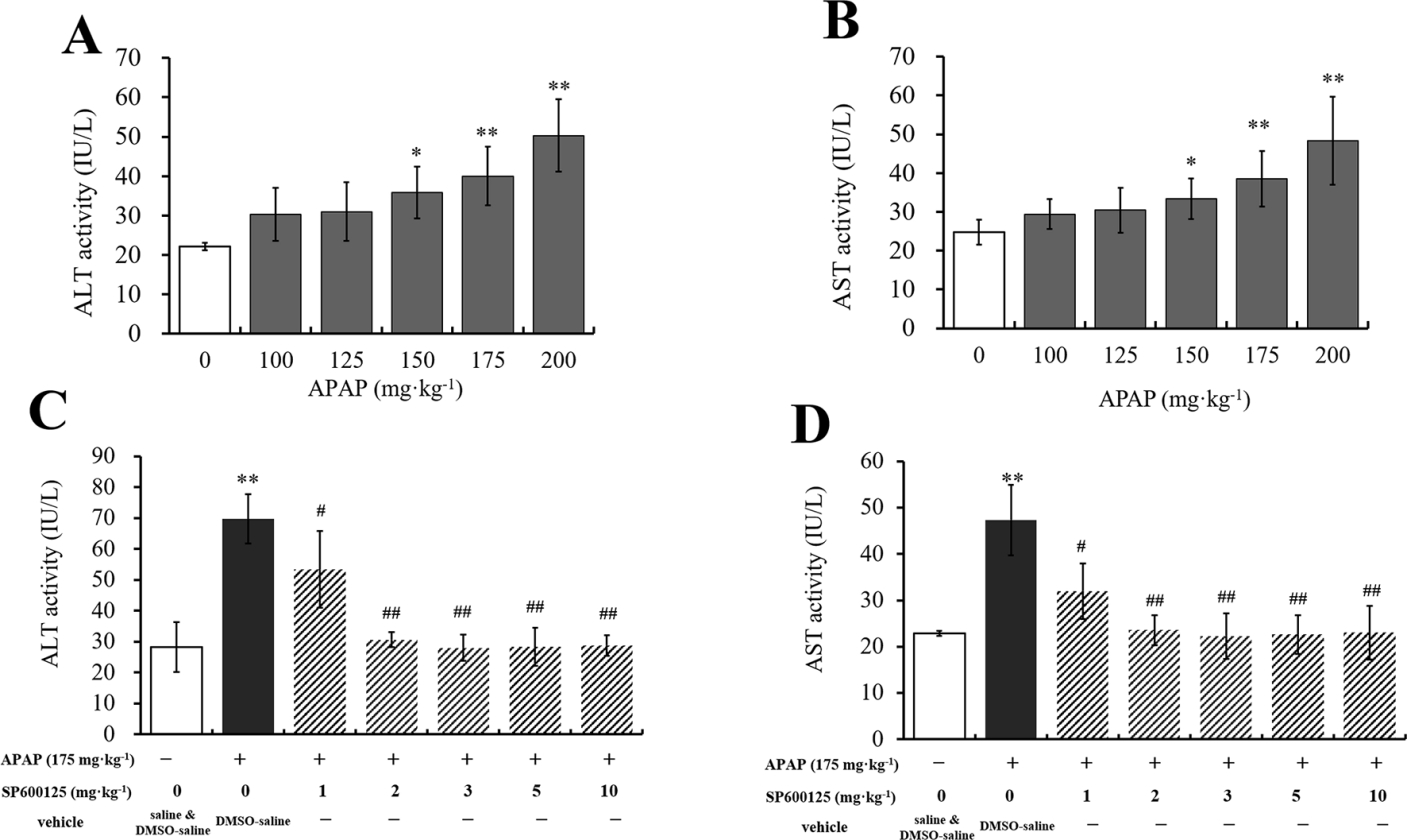
Dosage selection of APAP and SP600125. **(A**, **B)** Changes of ALT and AST activities with different dosages of APAP in serum of mice. **(C**, **D)** Changes of ALT and AST activities with 175 mg·kg^−1^ APAP and different dosages of SP600125 in serum of mice. Values represented as means ± SD, n = 6. *Represents statistical differences caused by APAP. ^#^Represents statistical differences caused by SP600125 under the same dose of APAP. 0.05 > *P* > 0.01 (*, ^#^). *P* < 0.01 (**, ^##^).

To select the proper doses of APAP that can cause different degrees of hepatic injury, we measured serum ALT and AST activities, which are the special traditional biomarkers of liver damage. It was observed that ALT and AST activities were gradually increased with the increasing doses of APAP administration (**Figures 1A, B**). Compared to vehicle group, ALT and AST activities in 150 mg·kg^−1^ APAP group were markedly increased (*P* < 0.05), and those in 175 mg·kg^−1^ APAP group were significantly increased (*P* < 0.01). Thus, we chose 150 and 175 mg·kg^−1^ APAP for further study. Then, ALT and AST activities of mice administered with SP600125 and APAP were tested to select the proper dosage of SP600125. It was observed that SP600125 ameliorated hepatic injury (**Figures 1C, D**). Compared with the model group, 1 mg·kg^−1^ SP600125 markedly decreased ALT and AST activities (*P* < 0.05), while SP600125 significantly decreased the activities (*P* < 0.01) of mice treated at a dose of 2 mg·kg^−1^ or higher. Thus, we chose 2 mg·kg^−1^ SP600125, due to little difference between these doses, for further study. Moreover, we evaluated whether SP600125 alone had effect on serum ALT and AST activities in mice. The results showed that there was no significant difference in ALT and AST activities of SP600125-treated (2 mg·kg^−1^) mice as compared to the DMSO–saline–treated mice (**Supplementary Figure 1**).

### JNK Signaling Pathway Activated by APAP

**Figure 2:**
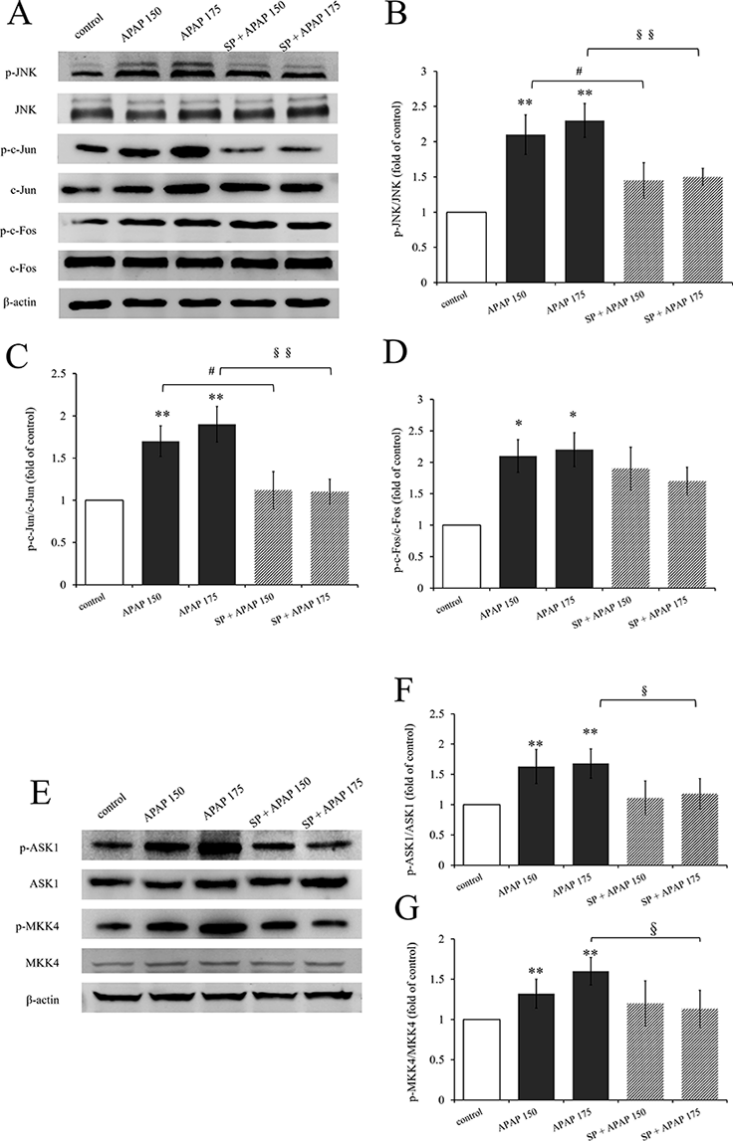
Activation of JNK signaling pathway under different dosages of APAP. **(A)** Western blot analyses of total tissue lysate for p-JNK, JNK, p-c-Jun, c-Jun, p-c-Fos, c-Fos, and β-actin (loading control). **(B**–**D**, **F**, **G)** Change of JNK activation (fold of p-JNK/JNK), c-Jun activation (fold of p-c-Jun/c-Jun), c-Fos activation (fold of p-c-Fos/c-Fos), ASK1 activation (fold of p-ASK1/ASK1), and MKK4 activation (fold of p-MKK4/MKK4) under different dosages of APAP in liver of mice with or without SP600125. **(E)** Western blot analyses of total tissue lysate for p-ASK1, ASK1, p-MKK4, MKK4, and β-actin (loading control). Values represented as means ± SD, n = 3. *Represents statistical differences caused by APAP. ^#^Represents statistical differences caused by SP600125 under 150 mg·kg^−1^ APAP; §Represents statistical differences caused by SP600125 under 175 mg·kg^−1^ APAP. 0.05 > *P* > 0.01 (*, ^#^, ^§^). *P* < 0.01 (**, ^§§^).

Expression levels of proteins related to JNK signaling by Western blot are represented in **Figure 2**. The activation levels of JNK (ratio of p-JNK/JNK, **Figure 2B**) and c-Jun (ratio of p-c-Jun/c-Jun, **Figure 2C**) in 150 mg·kg^−1^ APAP group and 175 mg·kg^−1^ APAP group were significantly increased (*P* < 0.01), which indicated that JNK signaling pathway was activated in APAP-induced hepatotoxicity, even at a lower dose. C-Jun N-terminal kinase inhibitor SP600125 significantly decreased JNK activation with administration of 175 mg·kg^−1^ APAP (*P* < 0.01) and markedly decreased with administration of 150 mg·kg^−1^ APAP (*P* < 0.05). Interestingly, the degree of JNK down-regulation by SP600125 was independent on the dosage of hepatotoxin (**Figures 2B, C**). **Figure 2D** shows the change of c-Fos activation under APAP and SP600125. There was no significant difference between SP600125 groups and APAP groups, although the APAP groups showed marked difference compared to the control group (*P* < 0.05).

### SP600125 Inhibited Upstream Proteins Activation of JNK Signaling Pathway

In addition, we detected the activation of ASK1 and MKK4, upstream proteins of JNK signaling pathway. The activation levels of ASK1 (ratio of p-ASK1/ASK1, **Figure 2F**) and MKK4 (ratio of p-MKK4/MKK4, **Figure 2G**) in 150 mg·kg^−1^ APAP group and 175 mg·kg^−1^ APAP group were significantly increased (*P* < 0.01) compared to the control group. But incredibly, SP600125 depressed their activation slightly, and there were even marked differences both on ASK1 and MKK4 activation between mice with or without JNK inhibitor at the dose of 175 mg·kg^−1^ APAP.

### JNK Inhibition Ameliorated APAP-Induced Liver Toxicity

**Figure 3:**
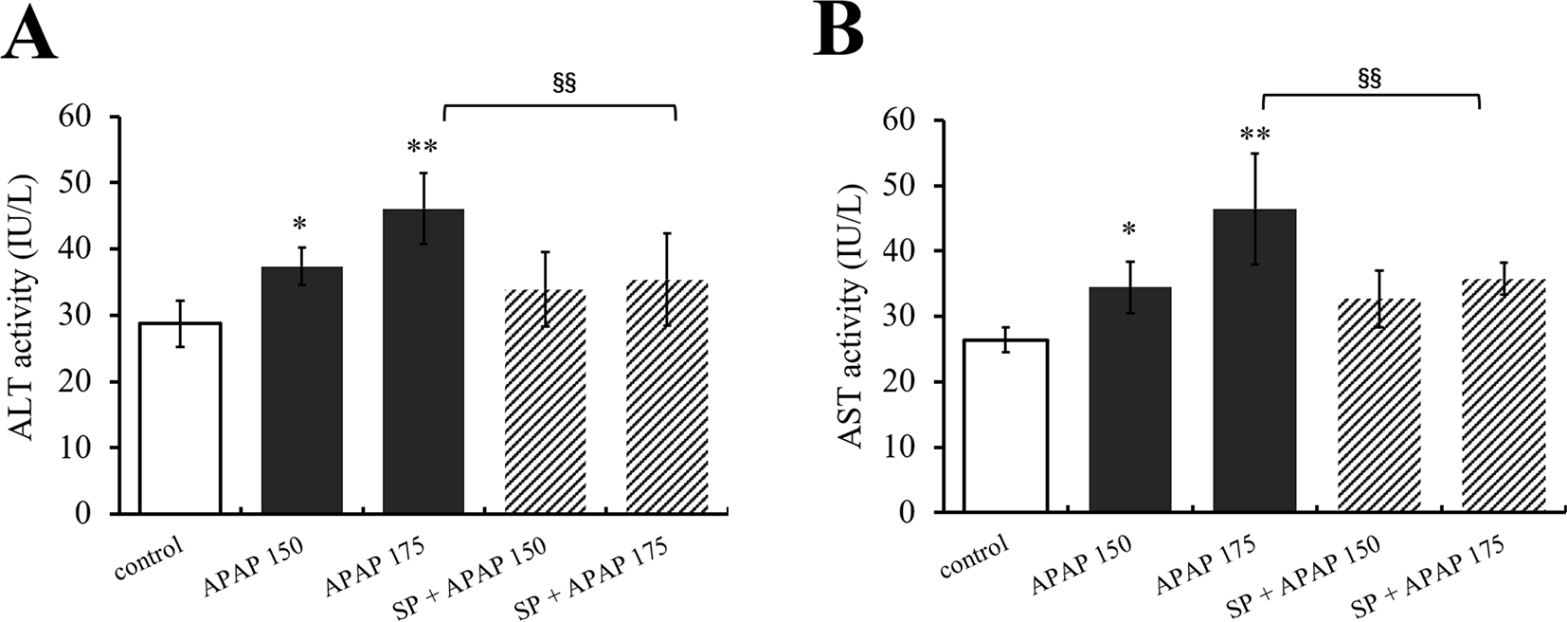
Inhibiting JNK ameliorated APAP-induced liver toxicity respect to ALT and AST. **(A)** Change of ALT activity under different dosages of APAP in serum of mice with or without SP600125. **(B)** Change of AST activity under different dosages of APAP in serum of mice with or without SP600125. Values represented as means ± SD, n = 6. *Represents statistical differences caused by APAP. ^§^Represents statistical differences caused by SP600125 under the same dose of APAP. 0.05 > *P* > 0.01 (*). *P* < 0.01 (**, ^§§^).

To clarify the effect of JNK signaling on APAP-induced hepatotoxicity, ALT and AST activities were measured, and the results are represented in **Figure 3**. Alanine aminotransferase and AST activities of mice administered with 150 mg·kg^−1^ APAP were markedly increased (*P* < 0.05, **Figures 3A, B**), and ALT and AST activities in 175 mg·kg^−1^ APAP group were significantly increased compared with the control group (*P* < 0.01). However, SP600125 significantly decreased the activities with 175 mg·kg^−1^ APAP (*P* < 0.01), indicating that inhibiting JNK depressed high-dose APAP-induced liver injury.

**Figure 4:**
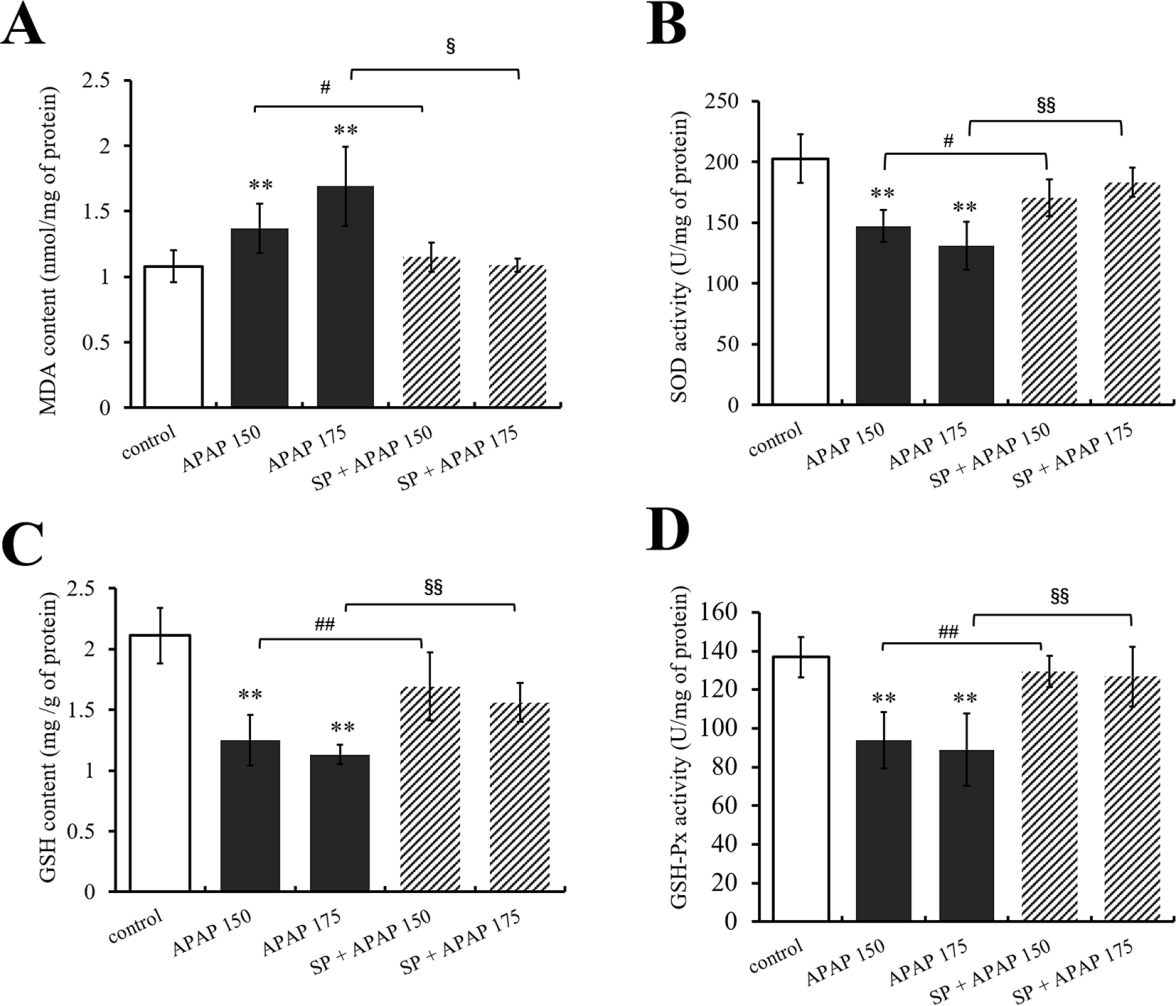
Changes of oxidative stress under different dosages of APAP in liver of mice with or without SP600125. **(A)** Change of MDA content under different dosages of APAP in liver of mice with or without SP600125. **(B)** Change of SOD activity under different dosages of APAP in liver of mice with or without SP600125. **(C)** Change of GSH content under different dosages of APAP in liver of mice with or without SP600125. **(D)** Change of GSH-Px activity under different dosages of APAP in liver of mice with or without SP600125. Values represented as means ± SD, n = 6. *Represents statistical differences caused by APAP. ^#^Represents statistical differences caused by SP600125 under 150 mg·kg^−1^ APAP. ^§^Represents statistical differences caused by SP600125 under 175 mg·kg^−1^ APAP. 0.05 > *P* > 0.01 (^#^, ^§^). *P* < 0.01 (**, ^##^, ^§§^).

Furthermore, the levels of GSH, GSH-Px, SOD, and MDA in liver were measured to evaluate the role of JNK in oxidative stress caused by APAP. As shown in **Figure 4A**, MDA content in liver was significantly (*P* < 0.01) increased under administration of 175 and 150 mg·kg^−1^ APAP compared with the control group, while it was markedly (*P* < 0.05) decreased at the presence of SP600125. Different dosages of APAP caused a significant decrease of SOD activity, GSH content, and GSH-Px activity in liver (*P* < 0.01, **Figures 4B–D**). SP600125 significantly increased SOD activity, GS H content, and GSH-Px activity when treated with 175 mg·kg^−1^ APAP. At a dose of 150 mg·kg^−1^ APAP, SP600125 increased GSH content and GSH-Px activity significantly (*P* < 0.01), and SOD activity markedly (*P* < 0.05) raised.

**Figure 5:**
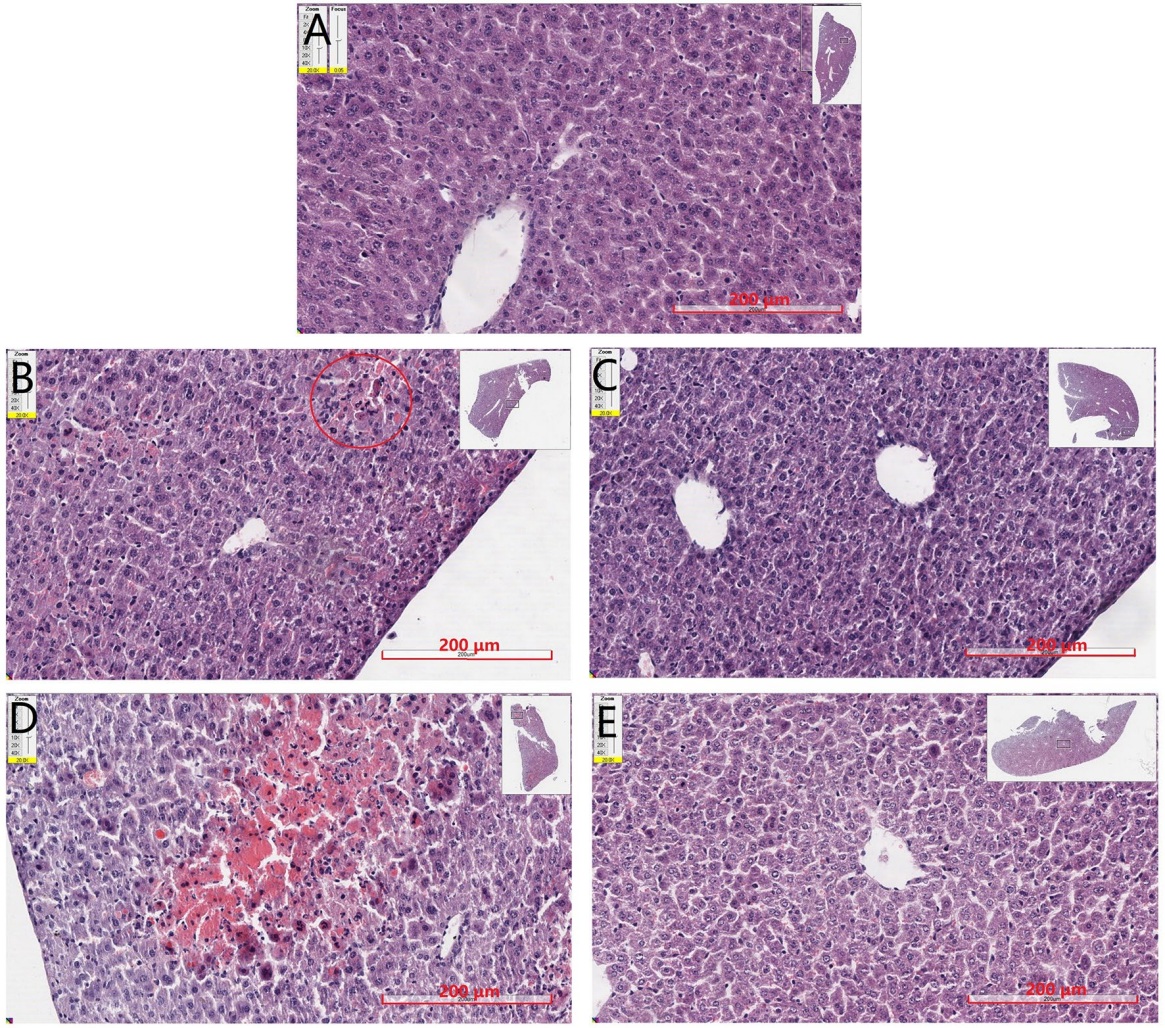
Mouse hepatic pathology. Liver sections were stained by HE, scanned by slide scanner, and the images are displayed at 200× magnification. **(A)** Mice from control group. **(B)** Mice treated with 150 mg·kg^−1^ APAP. **(C)** Mice treated with 150 mg·kg^−1^ APAP and SP600125. **(D)** Mice treated with 175 mg·kg^−1^ APAP. **(E)** Mice treated with 175 mg·kg^−1^ APAP and SP600125.

Liver tissues were examined by H&E staining, and histopathological micrographs are represented in **Figure 5**. Normal hepatic cells were observed in the control group with distinct lobuli hepatis (**Figure 5A**). After 150 mg·kg^−1^ APAP administration (**Figure 5B**), the cells appeared slight hepatic cord disorders and inflammatory cell infiltration with a few blutene chloride relative to the control group (**Figure 5A**). Mice treated with 175 mg·kg^−1^ APAP (**Figure 5D**) showed severe liver damage, inflammatory cell infiltration, necrocytosis, and hyperchromatic nuclei with congestion but no hepatic cord. Interestingly, in mice treated with SP600125 at 150 mg·kg^−1^ APAP (**Figure 5C**), it has been found that the hepatocytes showed normal morphology and appearance compared with 150 mg·kg^−1^ APAP group (**Figure 5B**). Liver photomicrographs of mice treated with JNK inhibitor and 175 mg·kg^−1^ APAP (**Figure 5E**) showed some inflammatory cells around central veins but little necrocytosis or hyperchromatic nuclei relative to the control group (**Figure 5A**).

### Inhibition of JNK Ameliorated APAP-Induced Apoptosis

**Figure 6:**
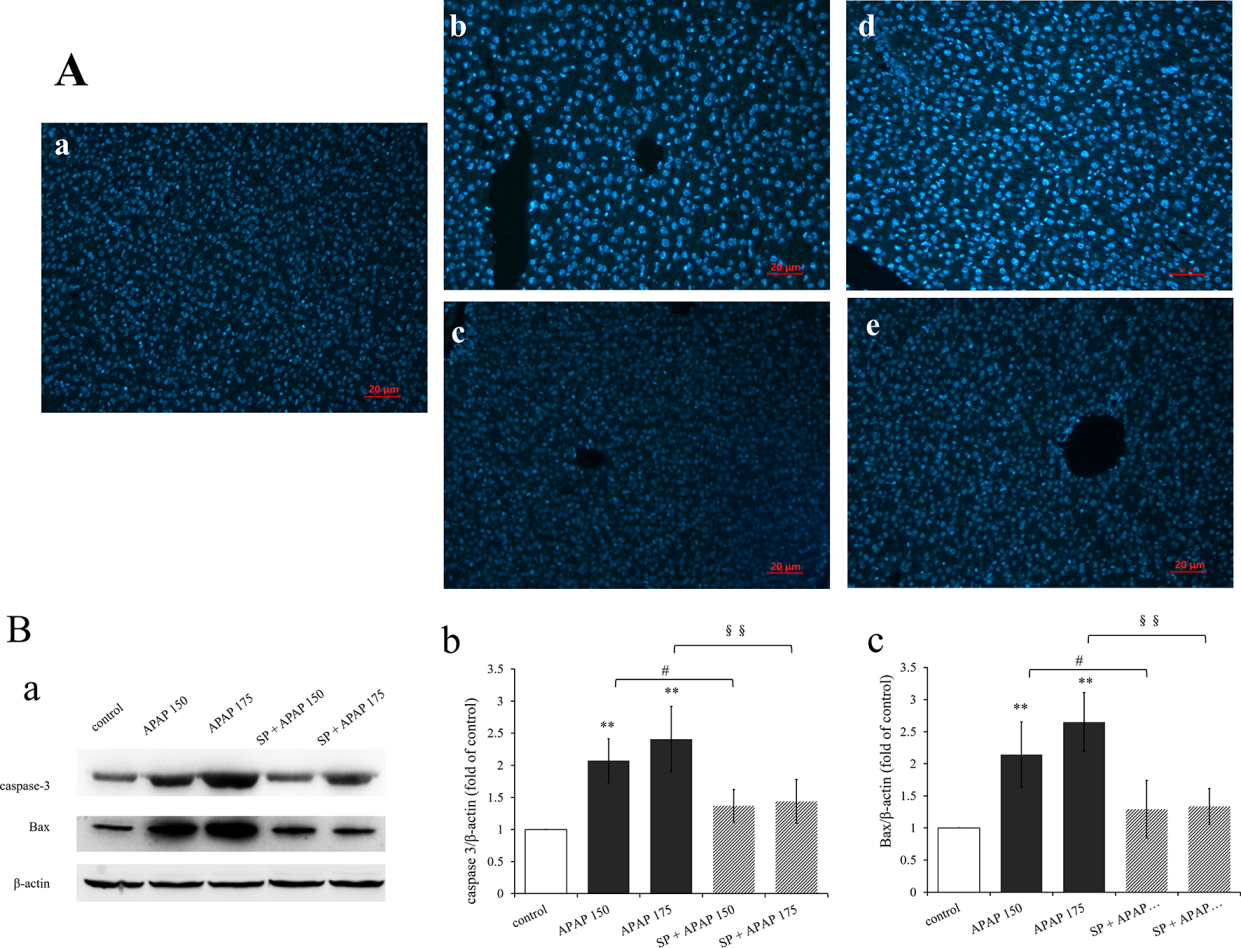
Inhibition of JNK ameliorated APAP induced apoptosis. **(A)** Liver sections were stained by Hoechst 33258, examined by fluorescence microscope and the images are displayed at 200× magnification. **(a)** Mice from control group. **(b)** Mice treated with 150 mg·kg^−1^ APAP. **(c)** Mice treated with 150 mg·kg^−1^ APAP and SP600125. **(d)** Mice treated with 175 mg·kg^−1^ APAP. **(e)** Mice treated with 175 mg·kg^−1^ APAP and SP600125. **(B)** Western blot analyses of total tissue lysate for caspase-3, Bax and β-actin (loading control), n = 3. **(a)** Immunoblot graph of caspase-3 Bax and β-actin. **(b**, **c)** Changes of caspase-3 expression (fold of GSTA1/β-actin) and Bax expression (fold of Bax/β-actin) under different dosages of APAP in liver of mice with or without SP600125, n = 3. Values represented as means ± SD. *Represents statistical differences caused by APAP. ^#^Represents statistical differences caused by SP600125 under 150 mg·kg^−1^ APAP. ^§^Represents statistical differences caused by SP600125 under 175 mg·kg^−1^ APAP. 0.05 > *P* > 0.01 (^#^). *P* < 0.01 (**, ^§§^).

To confirm whether apoptosis participate in liver damage caused by different doses of APAP, apoptosis was detected by analyzing the nuclear morphology, which was evaluated with membrane-permeable blue Hoechst 33258. **Figure 6A** showed Hoechst 33258 staining fluorescence photomicrographs of liver sections. The fluorescence micrograph from control group (**Figure 6Aa**) showed few apoptotic cells, which was characterized by karyopyknosis. A few hepatocytes with small nuclei and nuclear pyknosis were seen in **Figure 6Ab** (150 mg·kg^−1^ APAP), and more nuclei of hepatocytes from mice treated with 175 mg·kg^−1^ APAP appeared hypercondensed, which was observed brightly stained (**Figure 6Ad**). The number of apoptotic cells induced by APAP was in a dose-dependent manner, with gradually increasing the number of nuclear pyknosis. However, apoptosis was significantly reduced by the administration of SP600125 both at 150 and 175 mg·kg^−1^ APAP dose (**Figures 6Ac, e**). Expression levels of proteins related to apoptosis by Western blot are represented in **Figure 6B**. The expression levels of caspase-3 and Bax in 150 mg·kg^−1^ APAP group and 175 mg·kg^−1^ APAP group were significantly increased (*P* < 0.01), and JNK inhibitor SP600125 markedly decreased the expression levels of these two apoptotic proteins in mice with administration of 150 mg·kg^−1^ APAP (*P* < 0.05) and significantly decreased with administration of 175 mg·kg^−1^ APAP (*P* < 0.01). These results proved that apoptosis was increased in APAP-induced liver injury, and SP600125 can attenuate apoptosis induced by APAP.

### Effects of Inhibiting JNK on GSTA1

**Figure 7:**
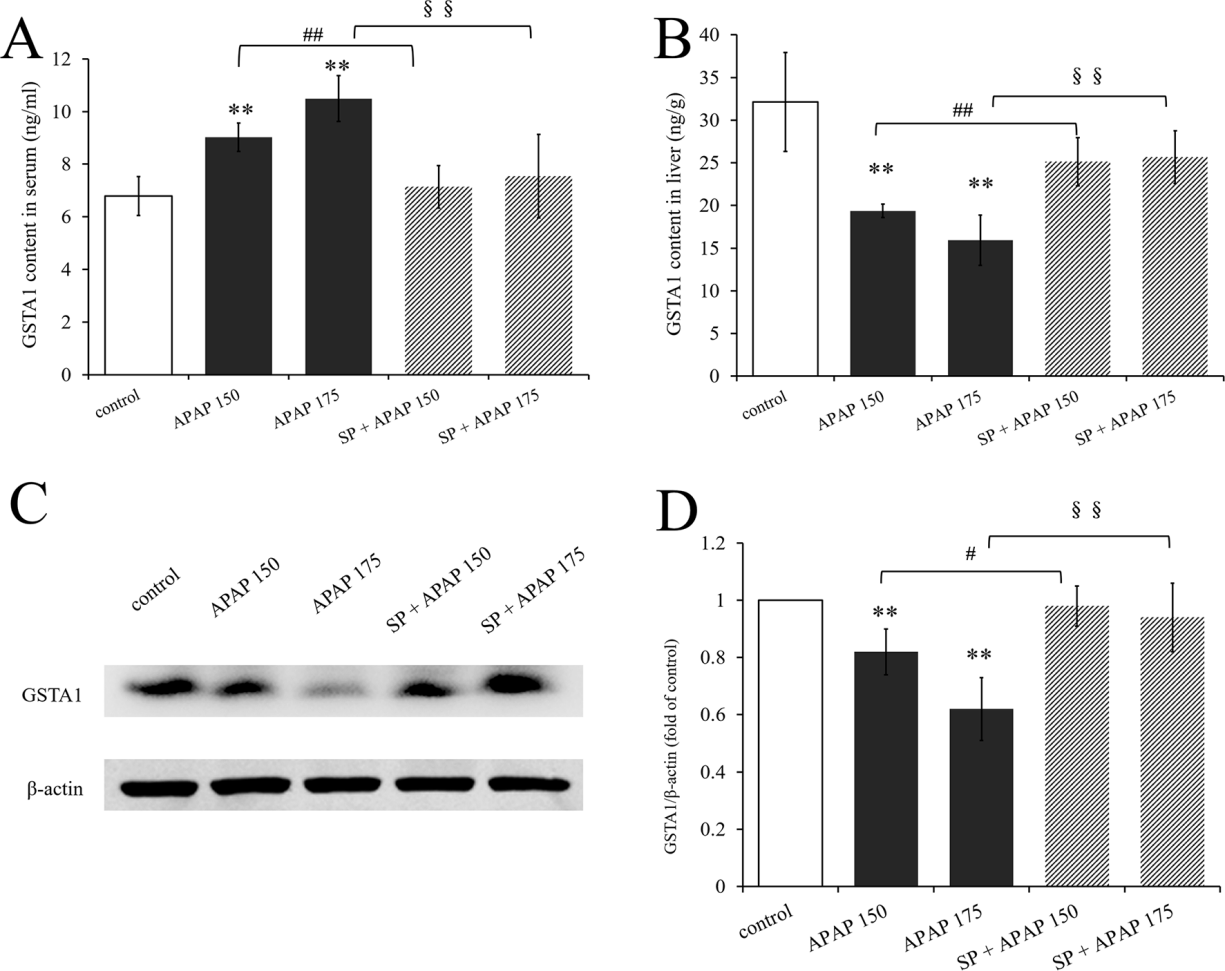
Changes of GSTA1 caused by different dosages of APAP in mice with or without SP600125. **(A)** Change of GSTA1 content under different dosages of APAP in serum of mice with or without SP600125, n = 6. **(B)** Change of GSTA1 content under different dosages of APAP in liver of mice with or without SP600125, n = 6. **(C)** Western blot analyses of total tissue lysate for GSTA1 and β-actin (loading control), n = 3. **(D)** Change of GSTA1 expression (fold of GSTA1/β-actin) under different dosages of APAP in liver of mice with or without SP600125, n = 3. Values represented as means ± SD. *Represents statistical differences caused by APAP. ^#^Represents statistical differences caused by SP600125 under 150 mg·kg^−1^ APAP. ^§^Represents statistical differences caused by SP600125 under 175 mg·kg^−1^ APAP. 0.05 > *P* > 0.01 (^#^). *P* < 0.01 (**, ^##^, ^§§^).

To investigate whether JNK signaling had effect on GSTA1 in hepatotoxicity, we determined GSTA1 content in serum and liver. As we can see, GSTA1 content in serum was significantly increased in two APAP groups, while that in liver was significantly decreased (*P* < 0.01, **Figures 7A, B**). However, SP600125 mitigated these changes both at the two dosages, and it was observed that GSTA1 content was significantly decreased in serum while increased in liver (*P* < 0.01) compared to the corresponding APAP group. In addition, we detected GSTA1 expression in liver by Western blot. **Figure 7C** is the immunoblot graph of GSTA1 and β-actin, and **Figure 7D** shows the densitometric analysis by ImageJ. The Western blot results showed that 175 and 150 mg·kg^−1^ APAP caused a significant decrease of GSTA1 expression (*P* < 0.01), but inhibiting JNK enhanced its expression significantly (*P* < 0.01) at a dose of 175 mg·kg^−1^ and markedly (*P* < 0.05) at a dose of 150 mg·kg^−1^. The results showed that the expression of GSTA1 decreased when mice were treated with APAP, but the expression level increased after inhibiting JNK signaling pathway. This indicated that JNK had effect on serum GSTA1 content and hepatic GSTA1 content and expression in APAP-induced liver damage.

## Discussion

Nowadays, with the rapid development of drug industry, people pay more and more attention to DILI (Zimmerman, 1981), which makes the study on DILI mechanism particularly important. Acetaminophen is widely used as antipyretic analgesic; however, APAP overdose is the leading cause of drug-induced liver failure, which makes it an ideal and practical model of DILI. It was first reported that the threshold for a single dose of APAP to induce hepatotoxicity in humans was estimated to be approximately 250 mg·kg^−1^ (Prescott, 1983). In addition, APAP over dosage could also cause liver damage or even necrosis in many species of laboratory animals, including rats, mice, hamsters, guinea pigs, rabbits, and dogs (Boyd and Bereczky, 1966; Mitchell et al., 1973; Davis et al., 1974; Gazzard et al., 1975). Mice are more commonly used than the other species, and this model is considered to be the golden model for studying the mechanisms for APAP-induced hepatotoxicity and searching for effective antidotes for liver injury. The present study was carried out with male mice because of their constant metabolism compared with the variations in the female physiology (Shi et al., 2017). It was reported that the dose for liver toxicity in mice was approximately 200 to 400 mg·kg^−1^ (Murray et al., 2015; Barman et al., 2016). In previous studies, the APAP solution was suspension, which caused a low repeatability (Ma et al., 2018). But in the present study, we dissolved APAP with saline at 40°C and treated mice with the solution. The results showed that administration of 150 mg·kg^−1^ APAP caused a marked increase of ALT and AST activities, while 175 mg·kg^−1^ APAP caused a significant increase. Activity of aminotransferase is a traditional biomarker of toxicity-mediated liver injury (Liu et al., 2014). Thus, the dosages at 150 and 175 mg·kg^−1^ were ascertained that could induce hepatic injury. Then, we investigated the role of the JNK signaling pathway in liver injury induced by different dosages of APAP.

C-Jun N-terminal kinase, also known as stress-activated protein kinase, plays an important role in various stress responses. It can be stimulated by growth factors, cytokines, some G-protein–coupled receptors, and environmental stress, including heat, radiation, hypoxia, ischemia-reperfusion, and hyperosmotic (Ip and Davis, 1998; Davis, 2000), regulating cellular proliferation, differentiation, survival, and apoptosis (Ip and Davis, 1998). Hepatic JNK activity was found a massive increase during APAP-induced liver failure in mice (Henderson et al., 2007). SP600125, a direct inhibitor of JNK1, 2, and 3, inhibits the activation of JNK by inhibiting phosphorylation of JNK in a dose-dependent manner (Yang et al., 2016a). Besides, it can inhibit the interactions between JNK and c-Jun by blocking the denosine triphosphate binding domain of JNK, thus preventing activation of the transcription factor c-Jun (Bennett et al., 2001; Shin et al., 2002). It was reported that low-dose APAP (150 mg·kg^−1^) caused mitochondrial depolarization and JNK activation, and SP600125 decreased mitochondrial depolarization (Hu et al., 2016). C-Jun N-terminal kinase is usually located in the cytoplasm, and it will translocate into the nucleus after phosphorylation. Then p-JNK could phosphorylate c-Jun and enhance its transcriptional activity, thereby regulating cell proliferation, differentiation, survival, and apoptosis (Ip and Davis, 1998). As we can see in **Figure 2**, JNK and c-Jun were phosphorylated at different dosages of APAP, and SP600125 reduced their activation significantly, indicating that JNK signaling pathway mediated different degrees of liver injury induced by different dosages of APAP. However, it was clear that the level of JNK phosphorylation caused by 150 mg·kg^−1^ APAP was not as high as that caused by 175 mg·kg^−1^ APAP. Interestingly, the levels of JNK phosphorylation inhibited by SP600125 were similar in the two dosages of APAP administration. Thus, we conjecture that the degree of JNK activation is positively correlated with the degree of liver injury, but the ability of SP600125 to specifically block JNK phosphorylation is not concerned with liver injury.

In addition, we measured the activation of c-Fos, reported as downstream of JNK signaling pathway (Yang et al., 2016b). Acetaminophen caused a marked increase on c-Fos activation, but SP600125 did not inhibit the activation. The results indicated that c-Fos is not the downstream protein of JNK signaling pathway, because its activation did not change with the change of JNK activation. Additionally, we measured activation of ASK1 and MKK4, which were reported as the upstream proteins of JNK (Win et al., 2018). The results showed that APAP significantly increased ASK1 and MKK4 activation, and there was a marked decrease under SP600125 administration at 175 mg·kg^−1^ APAP treatment. We wondered how JNK inhibitor could depress ASK1 and MKK4 phosphorylation. Apoptosis signal-regulating kinase 1 is a protein suited to maintain the homeostatic balance especially in the cell, as it is sensitive to ROS produced by NAPQI in the circumstance of APAP overdosage. In response to ROS, ASK1 becomes activated by homodimerization and autophosphorylation (Eaton, 2015), which will subsequently phosphorylate MKK4. In the present study, SP600125 decreased ASK1 and MKK4 activation slightly (ASK1 from 1.68 to 1.18, MKK4 from 1.60 to 1.13), while it decreased JNK activation strongly (from 2.33 to 1.48) at a dose of 175 mg·kg^−1^ APAP. We inferred that SP600125 protected mice from APAP-induced liver injury, and the level of ROS decreased, which contributed to depress ASK1 and MKK4 activation.

Transaminases like ALT and AST will be released from liver to plasma when hepatocytes are damaged, and traditionally, they are the most commonly used in the assessment of hepatic injury (Shi et al., 2017). In the present study, we measured ALT and AST activities by giving mice SP600125 to explore whether JNK signaling pathway mediates APAP-induced hepatic injury. Alanine aminotransferase and AST activities were significantly increased with 175 mg·kg^−1^ APAP, but SP600125 reduced the activities, which indicated that JNK participated in the process of APAP hepatotoxicity. Oxidative stress is caused by excessive ROS, the inactivation or the loss of antioxidant, or both (Schulz et al., 2000). Mitochondria and Kupffer cells are closely related to the oxidative stress that develops with APAP toxicity. Glutathione, as the first line of defense against free radicals, helps to counteract peroxidative damage in cells by binding to toxic metabolites and peroxides (Gaucher et al., 2018). Glutathione conjugates with NAPQI eventually eliminated in the urine when APAP is used at therapeutic dose. However, following an overdose of APAP, the hepatic GSH becomes depleted, and the toxic metabolite remains free to combine irreversibly with cellular proteins and to cause liver damage, necrosis, and death (Yan et al., 2018). Glutathione peroxidase protects cells by eliminating intracellular peroxides and peroxidation products (FlorholmenKjær et al., 2016). Superoxide dismutase is considered as antioxidant defense enzyme against the potential free radicals released by toxic that cause oxidative stress, which can inhibit the initiation of lipid peroxidation (Hsu et al., 2009). Malondialdehyde is one of lipid peroxidation products, which has been used as a biomarker of lipid peroxidation for several decades (Lykkesfeldt, 2007). In the present study, APAP caused significant increase of MDA content but decrease of GSH content, SOD, and GSH-Px activities, which indicated that there was severe oxidative stress in APAP-induced hepatotoxicity. Interestingly, administration of JNK inhibitor decreased MDA content in liver, indicating that SP600125 reduced lipid peroxidation; administration of JNK inhibitor increased GSH content, SOD and GSH-Px activities, indicating that SP600125 improved hepatocytes against free radicals and toxic metabolites. Thus, we can conclude that JNK signaling pathway is involved to oxidative stress induced by APAP.

The results of histopathological examination also demonstrated APAP-induced hepatotoxicity. Liver sections from the control group showed normal morphology and appearance. Pathological changes became more obvious with increasing dose of APAP. After SP600125 administration, it was found that inhibiting JNK reduced the liver damage caused by APAP. Previous studies demonstrated that toxic doses of APAP could induce apoptosis by activating caspase-3 and caspase-9 in rats (Abdel-Zaher et al., 2007; Ghosh and Sil, 2009). Understanding the molecular mechanism of JNK regulation of apoptosis can provide insights into the treatment and prevention of DILI. Experiment on PC12 cells has demonstrated that JNK signaling pathway is involved in the regulation of apoptosis caused by colistin, and JNK activation promotes apoptosis (Lu et al., 2017). In this study, apoptosis was detected by Hoechst 33258 staining, and expression levels of two key master apoptotic proteins, caspase-3, and Bax were analyzed by Western blot. The results showed that the apoptosis of liver cells became more apparent with the increasing dose of APAP. C-Jun N-terminal kinase inhibitor could significantly reduce apoptosis of hepatocytes induced by APAP. These results suggest that apoptosis exists during the process of APAP-induced liver injury, and SP600125 can attenuate apoptosis induced by APAP through the inhibition of JNK phosphorylation.

Glutathione S-transferases A1, as one of the phase II drug-metabolizing enzymes, protects cells from toxic and carcinogenic metabolites produced as a result of phase I drug-metabolizing enzymes (Prakash et al., 2015). It catalyzes the combination of GSH with NAPQI, which is the major toxic metabolite of APAP, and APAP-GSH adduct finally decreases GSTA1 activity in the liver. Previous studies of our group have demonstrated that GSTA1 is a more sensitive and accurate indicator than ALT and AST, and it could be released form liver to plasma when liver is damaged (Liu et al., 2014). It was reported that GSTA1 formed complexes with JNK in nonstressed cells. However, JNK signaling was activated following GSTA1-JNK complex dissociation under increasing ROS in Caco-2 cells (Adnan et al., 2012). In the present study, we noticed that APAP (150 and 175 mg·kg^−1^) decreased GSTA1 content in the liver and increased it in serum in a dose-dependent manner, indicating that GSTA1 was released from liver to plasma in liver damage caused by overdose of APAP. Interestingly, inhibition of JNK significantly increased hepatic GSTA1 content. Meanwhile, GSTA1 content was decreased in serum, which suggested that inhibiting JNK ameliorated liver damage, and JNK signaling pathway could affect GSTA1. Studies have demonstrated that GSTA1 suppresses activation of apoptotic JNK signaling by oxidative stress (Laura et al., 2006). Here, we want to know whether JNK signaling has any effects on GSTA1 expression. The results of Western blot showed that GSTA1 expression was reduced by APAP in a dose-dependent manner, indicating that expression of GSTA1 could reduce in liver damage. However, inhibiting JNK increased GSTA1 expression in mice administered with APAP (150 and 175 mg·kg^−1^). The data suggested that the activation of JNK suppressed GSTA1 expression in APAP-induced liver injury.

## Conclusion

Based on the above results, we can conclude that JNK signaling pathway could be activated in liver damage caused by different dosages of APAP. Inhibiting JNK signaling can attenuate the hepatic injury induced by APAP, depress the levels of oxidative stress and apoptosis, and reduce changes of GSTA1 content and expression caused by overdose APAP. These findings clearly demonstrated that JNK signaling pathway mediates APAP-induced hepatotoxicity involving oxidative stress and variations of GSTA1.

## Data Availability Statement

All datasets generated for this study are included in the manuscript/**Supplementary files**.

## Ethics Statement

The animal study was reviewed and approved by Harbin Veterinary Research Institute of Chinese Academy of Agricultural Sciences.

## Author Contributions

FL supervised the whole experiments. CS and BH performed the practical work and completed the experiments. CS wrote the whole manuscript. YY, YZ, YC, YL, CL, and RL provided help during the experiments. IM helped in improving language expression.

## Funding

This work was supported by the National Natural Science Foundation of China (grant 31472241).

## Conflict of Interest

The authors declare that the research was conducted in the absence of any commercial or financial relationships that could be construed as a potential conflict of interest.

## Abbreviations

ALT, alanine aminotransferase; APAP, acetaminophen; ASK1, apoptosis signal-regulating kinase 1; AST, aspartate aminotransferase; Bax, B cell lymphoma 2–associated x; caspase-3, cysteinyl aspartate specific protease 3; DILI, drug-induced liver injury; DMSO, dimethyl sulfoxide; GSH, glutathione; GSH-Px, glutathione peroxidase; GSTA1, glutathione S-Transferase alpha 1; JNK, c-Jun N-terminal kinase; MAPK, mitogen-activated protein kinase; MAP2K/MKK, mitogen-activated protein kinase kinase; MAP3K, mitogen-activated protein kinase kinase kinase; MDA, malondialdehyde; NAPQI, N-acetyl-p-benzoquinone imine; p-ASK1, phosphorylated apoptosis signal-regulating kinase 1; p-c-Fos, phosphorylated c-Fos; p-c-Jun, phosphorylated c-Jun; p-MKK4, phosphorylated mitogen-activated protein kinase kinase 4; p-JNK, phosphorylated c-Jun N-terminal kinase; ROS, reactive oxygen species; SOD, superoxide dismutase.
