# Supplementary material for: JNK Signaling Pathway Mediates Acetaminophen-Induced Hepatotoxicity Accompanied by Changes of Glutathione S-Transferase A1 Content and Expression
Source: Front Pharmacol. 2019 Sep 20;10:1092. doi: 10.3389/fphar.2019.01092 (PMC6763582; doi:10.3389/fphar.2019.01092)
Supplement: Supplementary file 1 [file DataSheet_1.docx]

Supplementary Material

# Supplementary Data


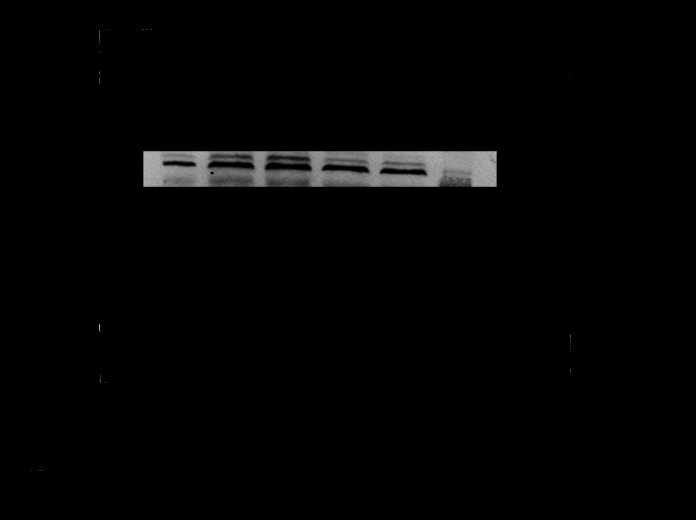


Image of Western Blot for p-JNK in Fig. 2.


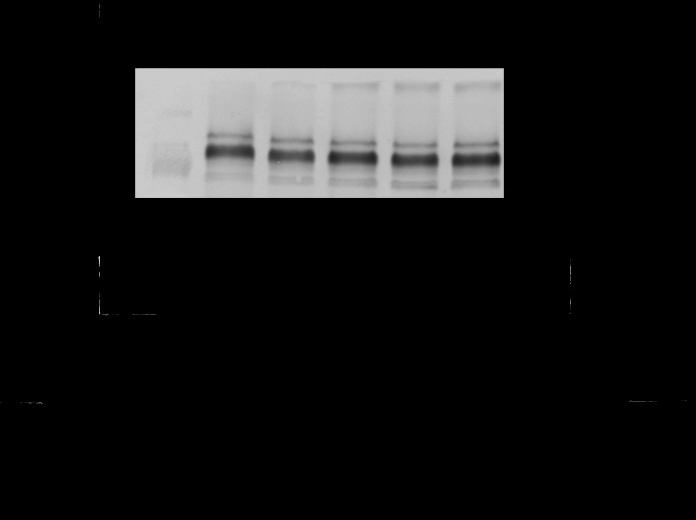


Image of Western Blot for JNK in Fig. 2.


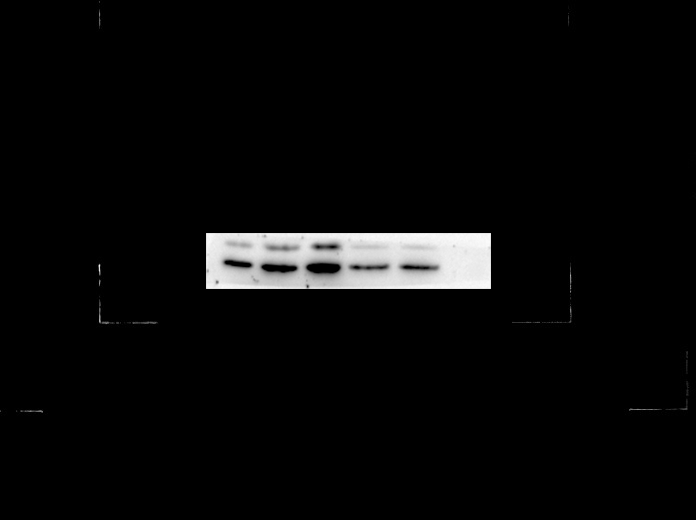


Image of Western Blot for p-c-Jun in Fig. 2.


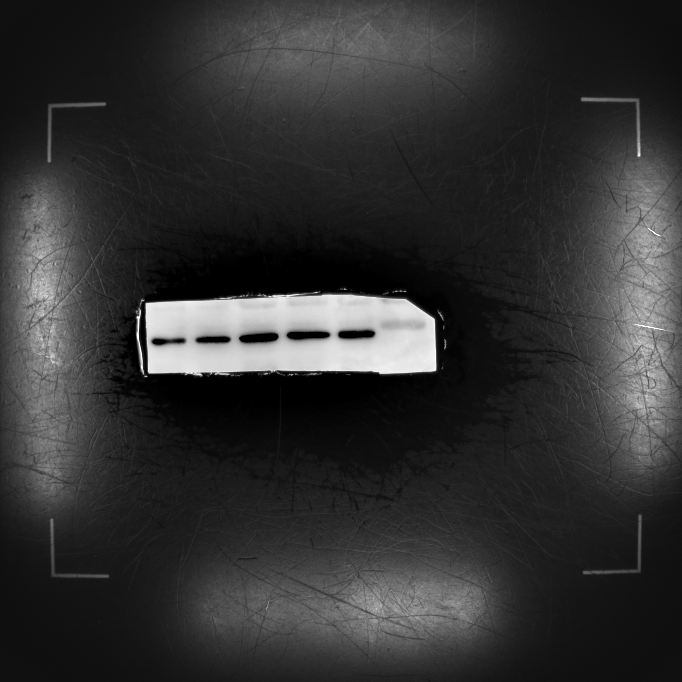


Image of Western Blot for c-Jun in Fig. 2.


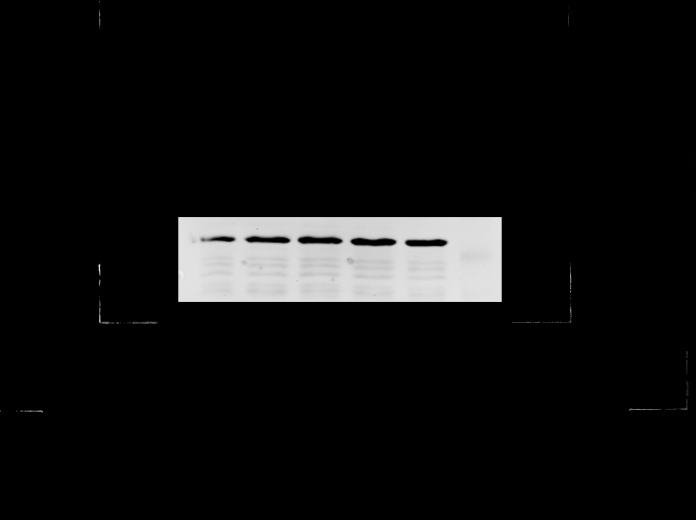


Image of Western Blot for p-c-Fos in Fig. 2.


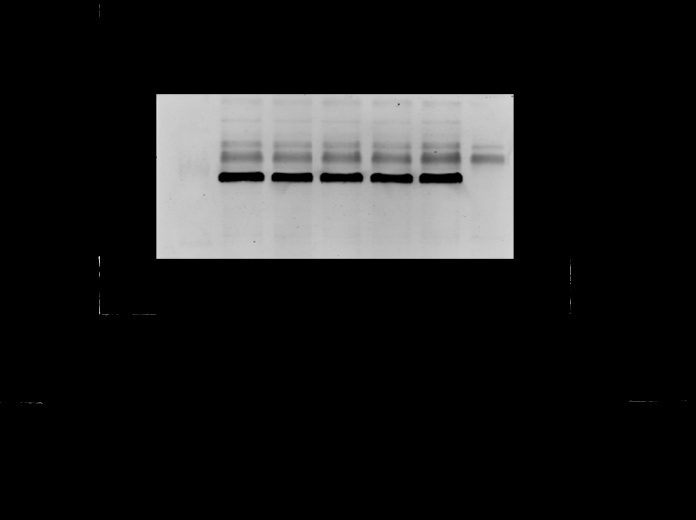


Image of Western Blot for c-Fos in Fig. 2.


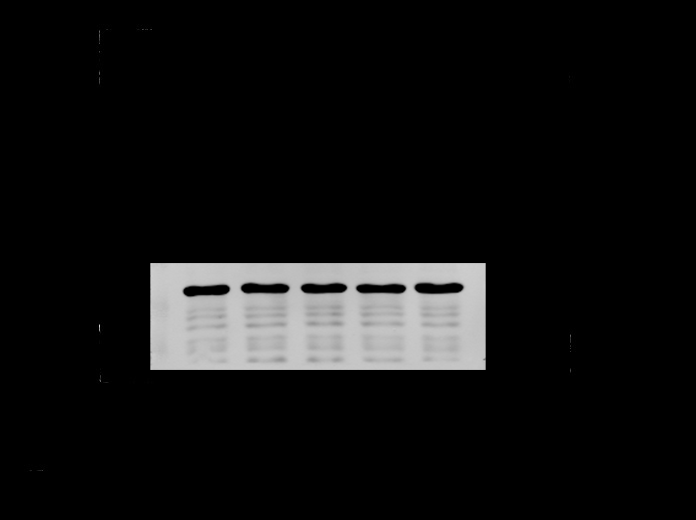


Image of Western Blot for β-actin in Fig. 2.


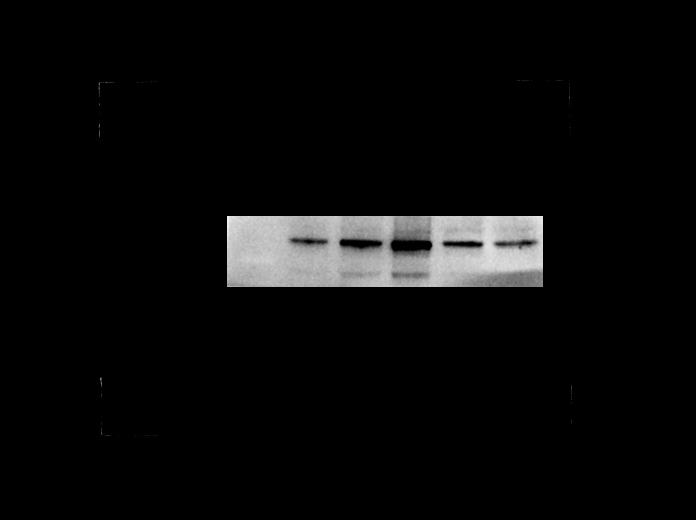


Image of Western Blot for p-ASK1 in Fig. 2.


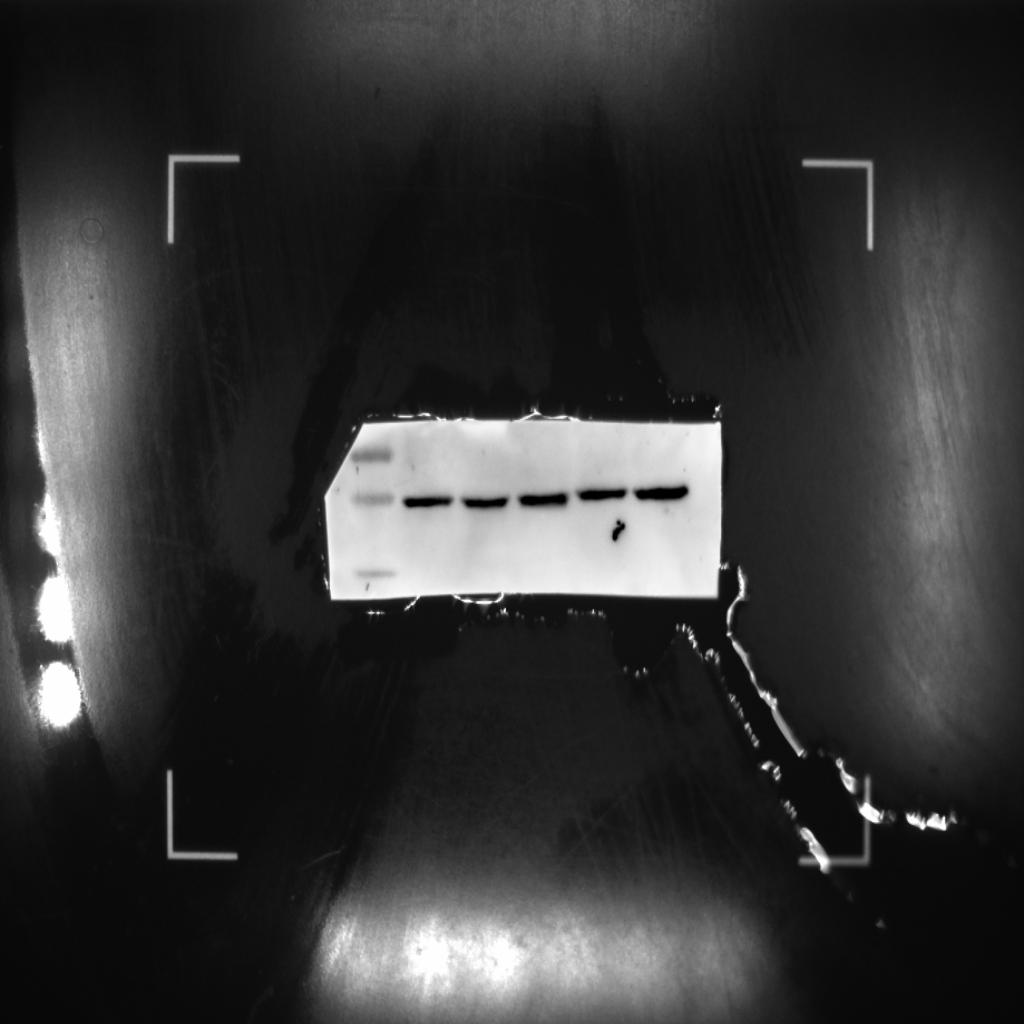


Image of Western Blot for ASK1 in Fig. 2.


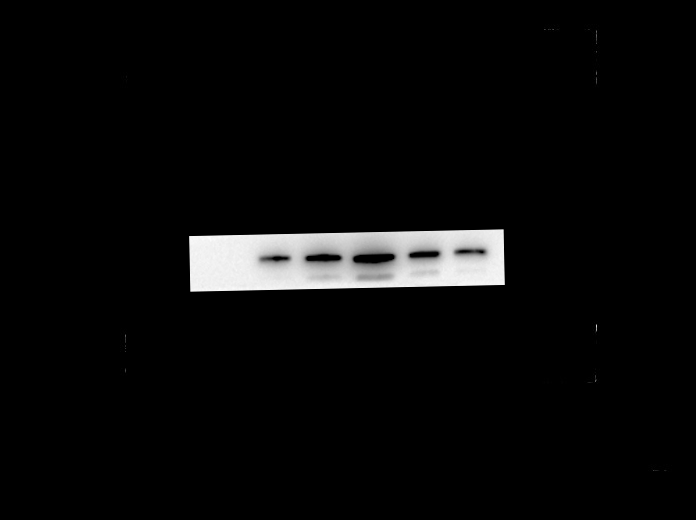


Image of Western Blot for p-MKK4 in Fig. 2.


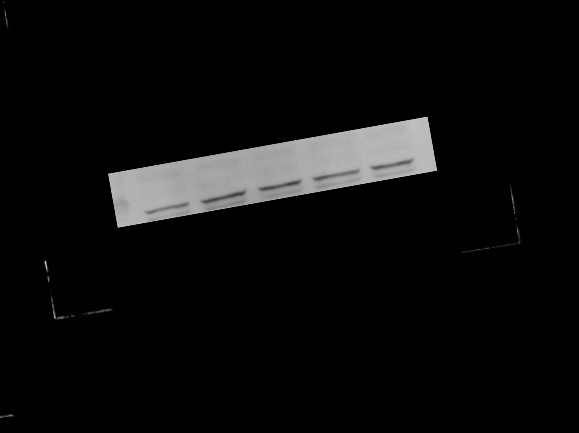


Image of Western Blot for MKK4 in Fig. 2.


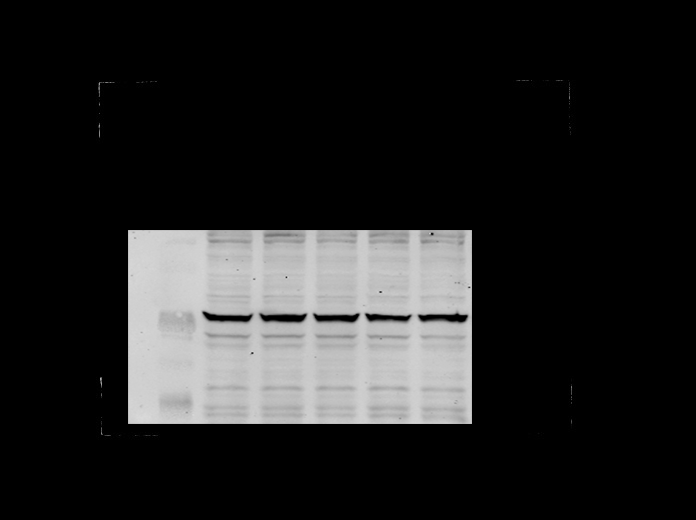


Image of Western Blot for β-actin in Fig. 2.


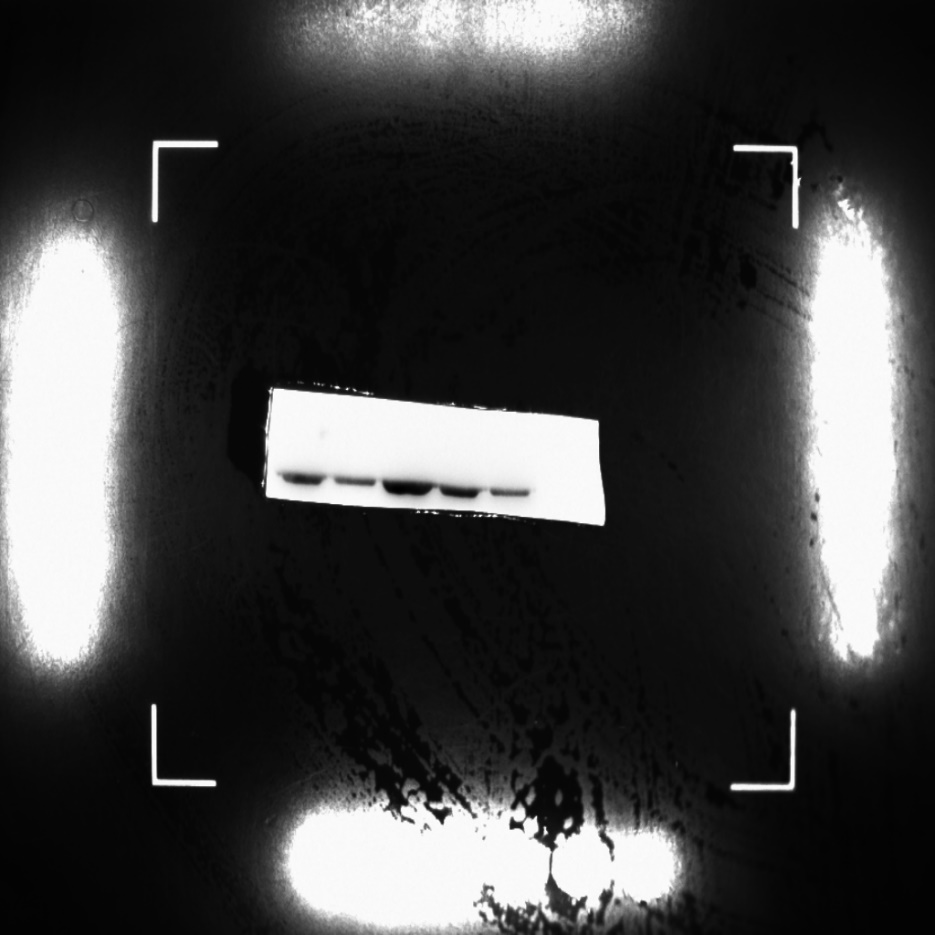


Image of Western Blot for caspase-3 in Fig. 6.


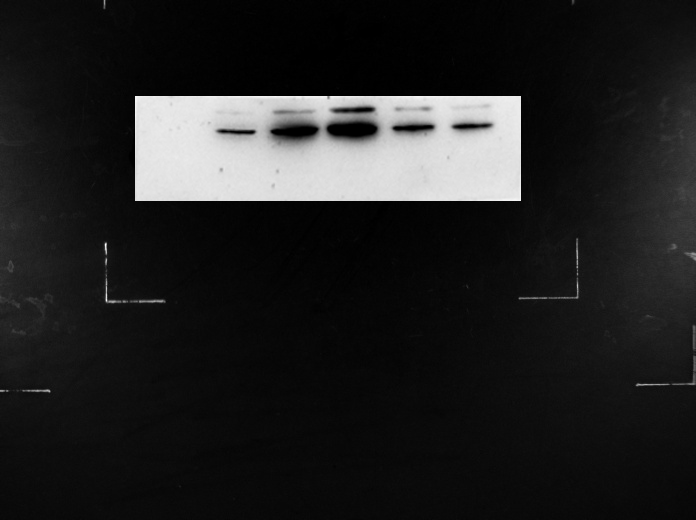


Image of Western Blot for Bax in Fig. 6.


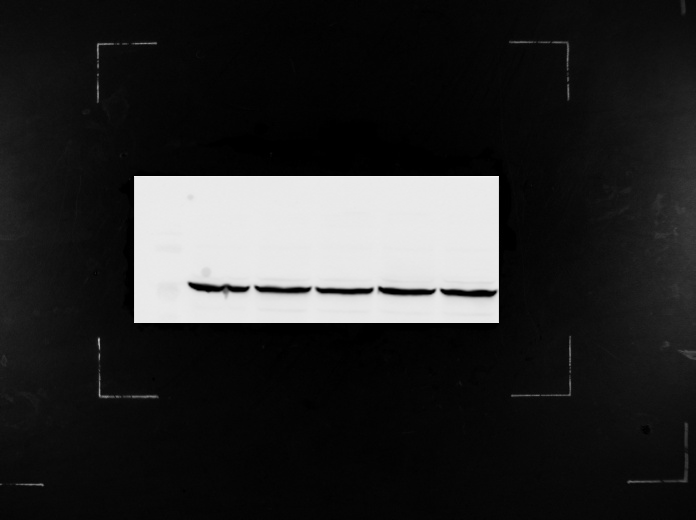


Image of Western Blot for β-actin in Fig. 6.


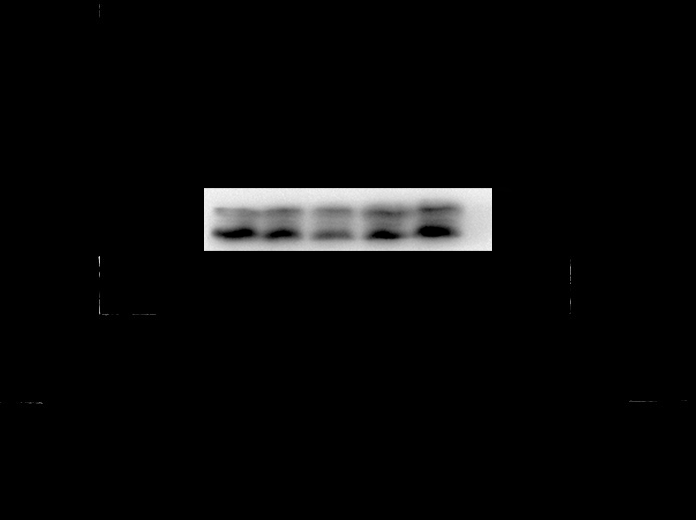


Image of Western Blot for GSTA1 in Fig. 7.


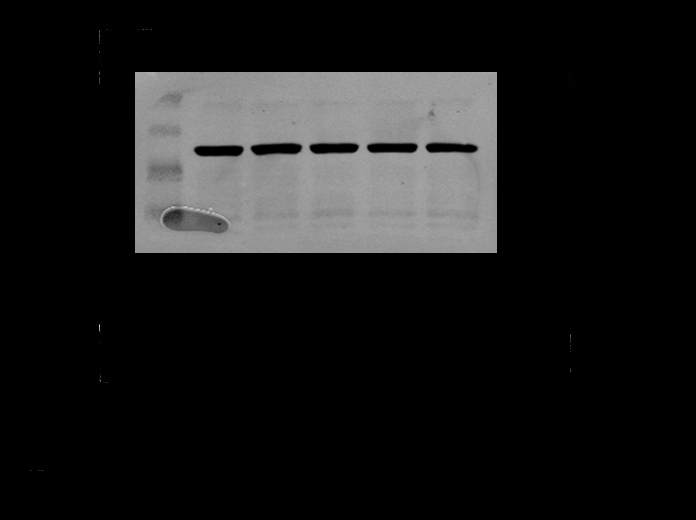


Image of Western Blot for β-actin in Fig. 7.

# Supplementary Figures and Tables


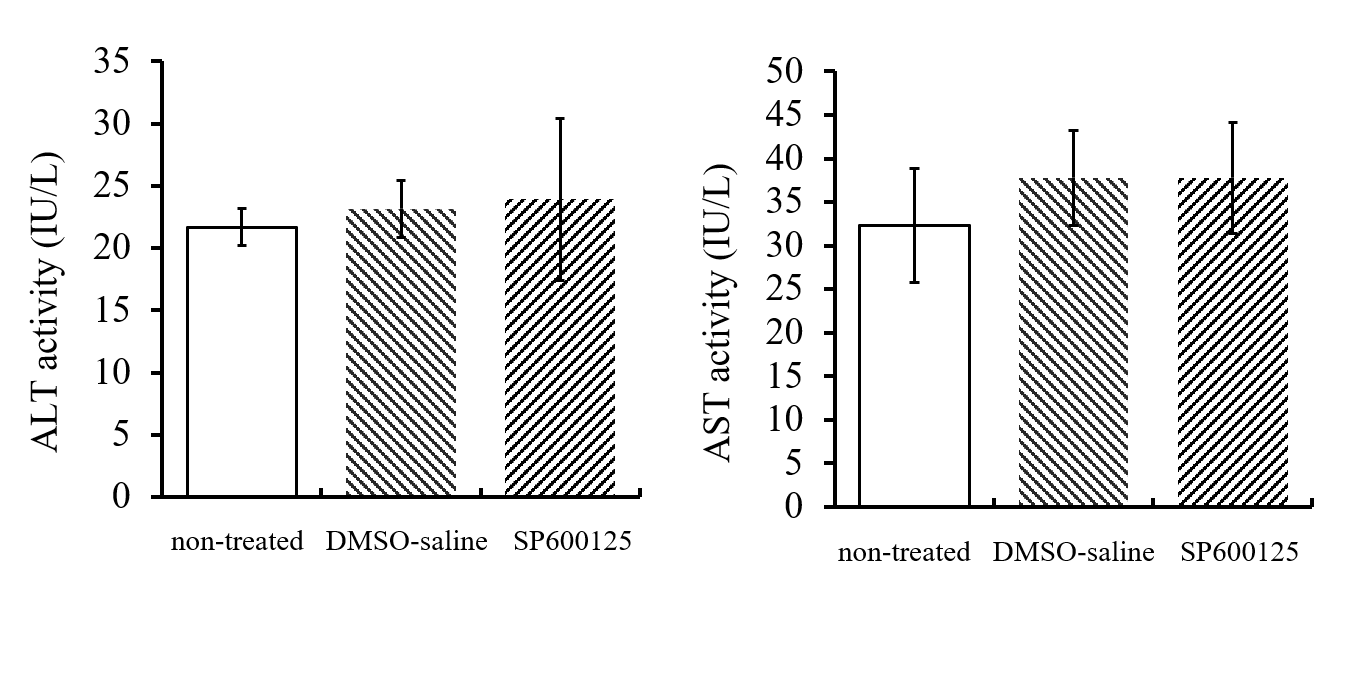


**Supplementary Figure 1.** Changes of ALT activity and AST activity with DMSO-saline or SP600125 in serum of mice. Values represented as means ± SD, n = 5.
